# Modelling Size-Dependent Cannibalism in Barramundi *Lates calcarifer*: Cannibalistic Polyphenism and Its Implication to Aquaculture

**DOI:** 10.1371/journal.pone.0082488

**Published:** 2013-12-12

**Authors:** Flavio F. Ribeiro, Jian G. Qin

**Affiliations:** School of Biological Sciences, Flinders University, Adelaide, South Australia, Australia; Institut Pluridisciplinaire Hubert Curien, France

## Abstract

This study quantified size-dependent cannibalism in barramundi *Lates calcarifer* through coupling a range of prey-predator pairs in a different range of fish sizes. Predictive models were developed using morphological traits with the alterative assumption of cannibalistic polyphenism. Predictive models were validated with the data from trials where cannibals were challenged with progressing increments of prey sizes. The experimental observations showed that cannibals of 25–131 mm total length could ingest the conspecific prey of 78–72% cannibal length. In the validation test, all predictive models underestimate the maximum ingestible prey size for cannibals of a similar size range. However, the model based on the maximal mouth width at opening closely matched the empirical observations, suggesting a certain degree of phenotypic plasticity of mouth size among cannibalistic individuals. Mouth size showed allometric growth comparing with body depth, resulting in a decreasing trend on the maximum size of ingestible prey as cannibals grow larger, which in parts explains why cannibalism in barramundi is frequently observed in the early developmental stage. Any barramundi has the potential to become a cannibal when the initial prey size was <50% of the cannibal body length, but fish could never become a cannibal when prey were >58% of their size, suggesting that 50% of size difference can be the threshold to initiate intracohort cannibalism in a barramundi population. Cannibalistic polyphenism was likely to occur in barramundi that had a cannibalistic history. An experienced cannibal would have a greater ability to stretch its mouth size to capture a much larger prey than the models predict. The awareness of cannibalistic polyphenism has important application in fish farming management to reduce cannibalism.

## Introduction

Polymorphism, the occurrence of discrete intraspecific morphs, is triggered by genetic differences, phenotypic plasticity, or a combination of both [1], [2]. In fish such as Arctic charr *Salvelinus alpinus* distinct intraspecific morphotypes can be a result of phenotypic plasticity associated with adaption to resources and ecological environments [3], [4]. Polyphenism on the other hand refers to alternative phenotypes in a population that are originated from a single genotype in response to environmental stimuli [2], [5]–[7]. If such phenotypic plasticity gives advantages for some individuals to ingest a larger prey and consume their conspecifics, this phenomenon is regarded as cannibalistic polyphenism.

True cannibalistic polyphenic individuals are clearly specialized in an intraspecific diet and have distinctive behaviour, morphology and life history [8], which are not common in fishes, but occur quite frequently in other taxa, such as amphibians [9]. Nonetheless, resource polymorphism has been reported in certain fish species [2]. For example, some individuals of Arctic charr exhibit a broader or larger mouth, faster growth rates and more aggressive behaviour than others [10]. In aquaculture, these traits are selected for, thereby leading to inadvertent selection of cannibalism in a farmed fish population [11], and causing frequent occurrence of intracohort cannibalism in piscivorous species. Furthermore, aquaculture conditions enhance the propensity of some individuals to become cannibals due to restriction of fish dispersing, overcrowding, and uneven food distribution, leading to size heterogeneity and cannibalism [11]–[13]. As a result, such conditions promote development of cannibalistic polyphenism.

The onset of intracohort cannibalism may occur shortly after hatch such as in dorada *Brycon moorei* [14], or at a later stage as in most marine fish [13] depending on the development patterns of the species. Once the cannibalistic process starts, it may persist during the juvenile phase of development as long as enough size heterogeneity enables a cannibal to prey on smaller conspecifics [13]. The current practice to control intracohort cannibalism in aquaculture is by size grading [11], [15], but such procedure is labour consuming, inefficient and stressful to fish [16]. As in the prey-predator relationship of teleosts, morphological factors determine the maximum prey size that predators can ingest [17]. Assuming that a cannibal can ingest a prey if the largest body dimension of the prey cross section is equal to or smaller than the maximum mouth dimension of the cannibal, some morphological models have been used to determine the largest size variation that is acceptable so as to make the exercise of complete cannibalism impossible after size sorting [18]–[22]. The largest prey cross-sectional dimensions (e.g., head height, body depth or width) are reliable factors for estimating the maximum capacity of a cannibal to ingest its prey. Nevertheless, the maximum mouth dimension may be subjective by researchers' choice [22]. Gape size [23], opened mouth height [15], [24], closed mouth width [22], [25], and opened mouth width [19]–[21], [26]–[28] have been used to predict the maximum ingestion capacity of cannibalistic fish species. However, in order to have a reliable prediction, the maximum mouth dimension must be carefully selected according to specific traits of the target species such as using mouth elasticity in snakehead *Channa striatus* [21] and orientation of the prey on cannibal mouth in orange-spotted grouper *Epinephelus coioides* [19] and giant grouper *E. lanceolatus* [20]. Furthermore, cannibalistic polyphenism has never been built into a model to predict size-dependent cannibalism in fish. As some individuals may possess larger jaws and a wider mouth [8], existing models based on the parts of a population average may underestimate the maximum prey size that a cannibal can ingest. Moreover, few models have been validated with an independent dataset, but if done, the maximum size of ingestible prey is underestimated as in snakehead [21] and largemouth bass *Micropterus salmoides* [22], or overestimated as in the giant [20] and orange-spotted [29] groupers.

The aim of this study was to determine size-dependent cannibalism in a highly cannibalistic fish, the barramundi *Lates calcarifer* (Latidae). Models were developed using the mouth width as the largest mouth dimension and the alternative assumption of polyphenism. Subsequently, the models were validated based on empirical results taken from a series of independent observations from different prey-predator pairs. Barramundi were used as the model species because it is an economically important fish for aquaculture in tropical and subtropical regions [16]. In a previous model, Parazo et al. [15] suggests that the total length (TL) of ingestible prey ranges 67–61% of the cannibal size in barramundi of 10–50 mm TL. However, Parazo's model was based on an inappropriate measurement of mouth size and the empirical validation might be prejudiced by prey size preference, as it was based on the stomach analysis of cannibals from an undisturbed population of cultured fish. Thus, the present study used a new approach to assess the maximum prey size that cannibalistic barramundi can ingest from direct observations. The new model simulates a more realistic scenario to quantify the size relationship between cannibal and victim individuals in cannibalistic fishes.

## Materials and Methods

### Ethics Statement

This study was carried out in strict accordance with the recommendations in the Animal Welfare Act 1985 and the Australian Code of Practice for the Care and Use of Animals for Scientific Purpose 7^th^ Edition. The protocol, species, and number of animals used in this study were approved by the Flinders University Animal Welfare Committee (Project Number: E347). In any trial situations, each prey had an opportunity to avoid the predators in their cannibal challenge since we allocated more open space in each aquarium to facilitate prey escape. Euthanasia procedures were performed under overdose (43 mg l^−1^) of AQUI-S® (New Zealand Ltd). All fish handling were followed by light anesthesia (15 mg l^−1^) with AQUI-S, and all efforts were made to alleviate fish suffering.

### Fish and rearing conditions

Hatchery raised barramundi *Lates calcarifer* of 34 days after hatching from the same cohort were obtained from West Beach Hatchery, West Beach, South Australia, and transported to the Animal House, Flinders University. Upon arrival, all fish were visually graded into large, medium and small sizes, and stocked into three holding tanks (300 l) filled with freshwater. Each tank was equipped with an external biofilter and kept at 27–28°C. Fish were divided into three groups and fed at different rates with dry pellets (NRD® range, 400–2,000 µm; 55% protein, 9% lipid, INVE Ltd, Thailand). Group 1: 360 large fish (1.2 fish l^−1^) fed to satiation twice a day in order to produce large individuals to be used as cannibals; Group 2: 950 small fish (3.2 fish l^−1^) fed once a day at a restricted ration to produce a range of small fish sizes to be used as prey on the cannibal challenge experiment; and Group 3: 650 medium fish (2.2 fish l^−1^) fed twice a day under moderate feeding restriction in order to promote a range of fish sizes to be used for morphological measurements. Tanks were cleaned twice a day to remove unfed pellets, faeces and dead fish. Water parameters were daily checked and maintained at 27.83±0.19°C, 7.69±0.21 mg l^−1^ dissolved oxygen, 7.51±0.02 pH, and <0.5 mg l^−1^ ammonia and nitrite nitrogen. A photoperiod of 12L:12D was used at a light intensity of 350 Lux during the hours of light with abrupt transition between dark and light periods.

### Morphological models construction

Periodically, 368 juveniles were sampled from fish in Group 3 for morphological measurements. Fish were collected with a hand net, euthanized with overdosed AQUI-S (43 mg l^−1^, AQUI-S New Zealand Ltd) and immediately measured for total length (TL, mm), body depth (BD, mm) and mouth width (MW, mm) to the nearest 0.01 mm using a dissecting microscope or a digital caliper. Fish from 15 to 140 mm TL were sampled, as this comprised the size range corresponding to the time interval when intracohort cannibalism was intense in barramundi fingerling culture [30]. The selection of morphological parts for measurement was under these two assumptions: (1) cannibalistic barramundi swallow their conspecific prey in whole with head first [13]; (2) when cannibalistic barramundi ingest their conspecific prey, the maximal prey body depth was positioned laterally from side to side in the cannibal mouth. Such assumptions were used to predict the maximum prey size for barramundi cannibals from 35 to 140 mm TL. Total length (TL) was measured as the distance from the tip of the snout to the end of the caudal fin and body depth (BD) as the distance between the anterior edge of the dorsal fin and the bottom of the abdomen. Two measurements of mouth width were taken: mouth width at the close position (MWc) as the distance between the outer edges of the maxillary bones just beneath the eyes with the mouth closed; and mouth width at the open position (MWo) as the horizontal largest cross-section distance with the mouth fully stretched in an ellipse shape. With both mouth width measurements, an estimate of mouth width extension (MWE) for each fish was calculated as MWE (%MWc) = [(MWo − MWc)/MWc)]×100.

The morphological predictive models were developed assuming that a TL_cannibal_ can swallow a TL_prey_ if the BD_prey_ is equal to or smaller than the MW_cannibal_. The relationships between MW_cannibal_ vs. TL_cannibal_ and BD_prey_ vs. TL_prey_ were used to predict the maximum prey length (TL_prey_) for given sizes of cannibals (TL_cannibal_). Models were developed using four different estimates of mouth size: closed mouth width (MWc); maximum closed mouth width (MWcmax); opened mouth width (MWo); maximum opened mouth width (MWomax).

### Cannibal challenge

A series of single pairwise trials were performed to empirically observe the maximum conspecific prey size that a cannibalistic barramundi can ingest. Cannibals from 25 to 131 mm TL were individually challenged with single conspecific prey of known sizes, starting from 45% of cannibal TL. The system consisted of 20×6 l aquaria (20×20×25 cm) connected to a freshwater recirculation system equipped with a communal 200 l biofilter and set in the same experimental room as the holding tanks. Aquaria were cleaned daily to remove faeces. Water quality and physical parameters were kept the same as those in the holding tanks.

Initially, 20 potential cannibals were sampled from fish in Group 1, anesthetized (AQUI-S, 15 mg l^−1^), measured for TL and individually stocked into each aquarium. Then, potential prey were collected from fish in Group 2, anesthetized (AQUI-S, 15 mg l^−1^), measured for TL, individually selected and matched their respective cannibal. No food was provided during the trials. Predation was checked twice a day (0900 and 1700 h). In case of predation, the cannibal was re-measured in order to decide the next prey size to be offered, and a new prey larger than the previous one would be selected from Group 2, anesthetized (AQUI-S, 15 mg l^−1^), measured for TL and individually matched the same cannibal. This procedure was repeated progressively by increasing the prey size at about 5% per change according to prey size availability. As the maximum prey size approached to the maximum ingesting limit for cannibals, the incremental rate of the new prey size was reduced to about 2%. The morphological limit for cannibals was considered maximum when both cannibal and prey coexisted for over 4 days. In that occasion, both fish were measured and the cannibal was replaced by a larger one.

Successful predation events were considered completion when the prey had been fully swallowed and digested by the cannibal. Cannibals in the process of digesting prey were easily identified due to their extended belly. Such consideration avoided significant discrepancies on growth rate between cannibal and prey during the next pairing period. In some circumstances, the prey was dead on the bottom of the aquaria after having been discarded by the cannibals due to unsuccessful capture attempts. In those cases, a new prey of a similar size was paired with the same cannibal. If the cannibal would kill but not ingest the prey again, that prey size was considered the upper limit of the cannibal and the cannibal was replaced by a larger one.

### Statistical analysis

All absolute estimates for body parts were regressed against TL and an analysis of covariance (ANCOVA) was used to test for homogeneity of the regression slopes of the body depth (BD, mm) and mouth width (MW, mm) estimates using total length (TL, mm) as a covariate. Linear regression analysis was used to assess the independence between mouth width extension (MWE as %MWc) and closed (MWc) and opened (MWo) mouth widths (%TL). MWE was regressed against TL to determine the capacities of mouth width extension as fish grew. Pearson's correlation analysis was used to assess the strength of correlations. All predictive models based on morphological measurements for the maximum prey to cannibal size ratio enabling the occurrence of intracohort cannibalism were developed using simple linear regression analysis. The results from the cannibal challenge experiment were used to estimate a revised model for maximum prey size for cannibals based on the empirical data. The size of the first offered prey was compared between the successful versus non-successful cannibalistic pairs with T-test to identify the criteria for the initial prey-predator size ratio that would provoke cannibalism. All statistics were considered significant at *P*<0.05.

## Results

### Morphological models

**Figure 1:**
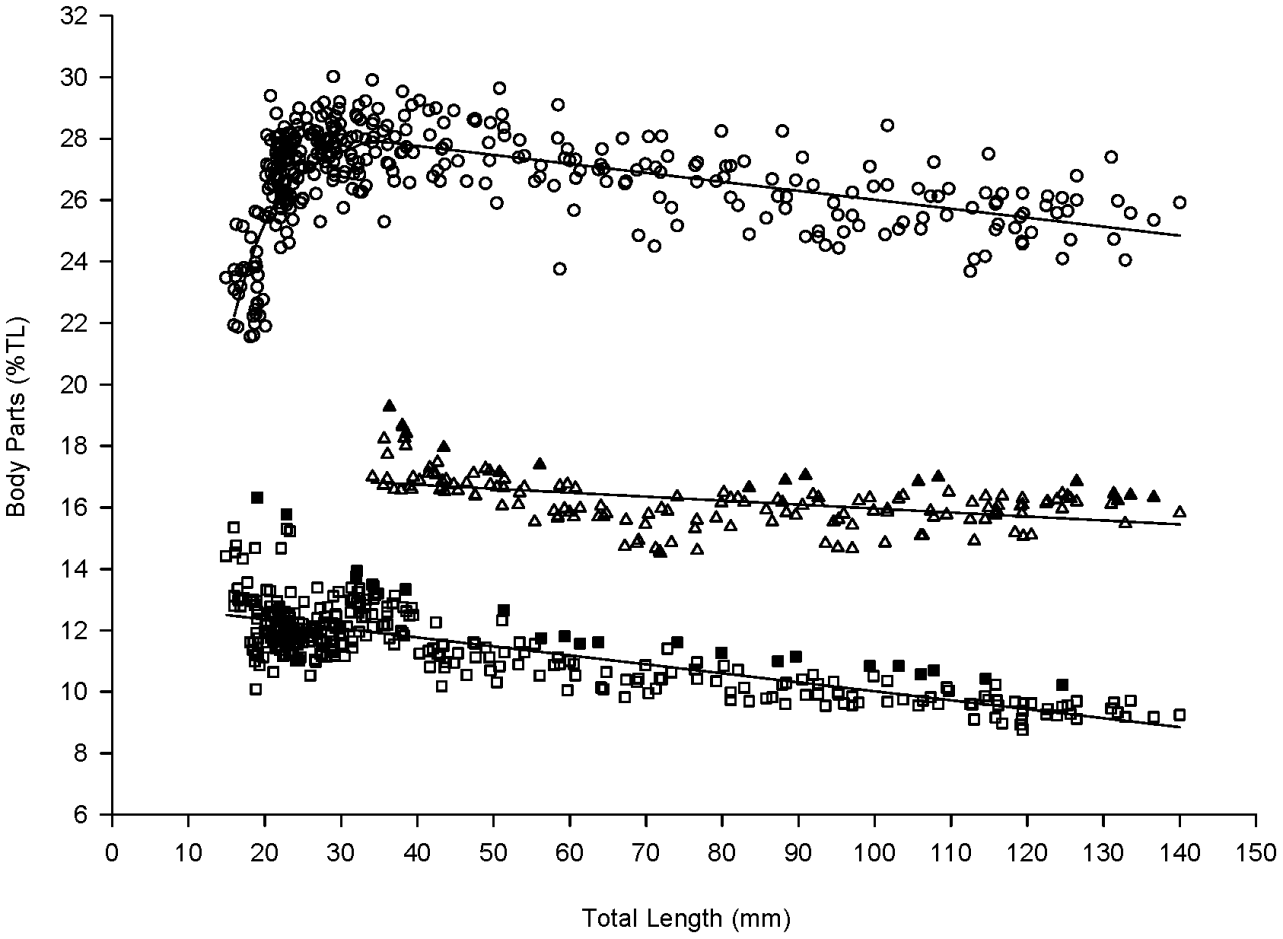
Morphological variation between relative body parts (%TL) and total length (TL, mm) of juvenile barramundi. Body depth (BD in circles, *n* = 368), closed mouth width (MWc in squares, *n* = 360) and opened mouth width (MWo in triangles, *n* = 154) are plotted against total length. Each symbol represents an individual estimate. Filled symbols represent maximum values of mouth width estimates.

During the early juvenile stage (15–30 mm TL), body depth (BD) showed positive allometric growth, attaining its maximum dimension relative to body size (28% TL) when fish were around 35 mm TL (Figure 1). Thereafter, BD slightly decreased and reached 25% TL at the late juvenile stage (140 mm TL; Figure 1). Mouth width at close (MWc) or open (MWo) presented slightly negative allometric growth as fish grew larger, decreasing from 13% to 9% TL (15–135 mm TL) and from 17% to 15% TL (35–135 mm TL), respectively (Figure 1).

**Figure 2:**
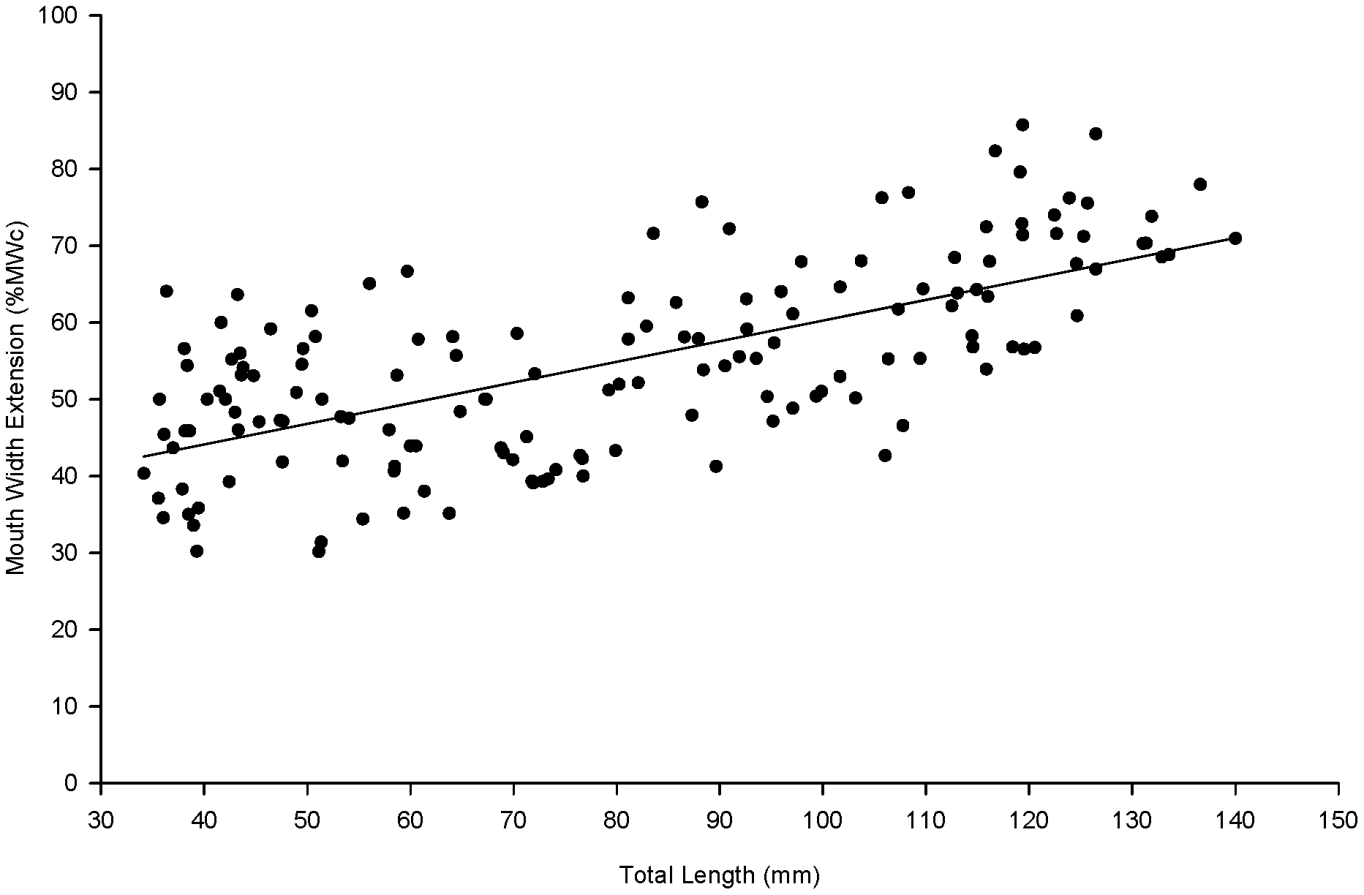
Relationship between mouth width extension (MWE, %MWc) and total length (TL, mm) of juvenile barramundi. MWE and TL are positive correlated (*r* = 0.660, *n* = 154, *P*<0.0001). The solid line represents the average MWE varying from 34 to 140 mm TL and is expressed as MWE = 0.269(0.024)TL + 33.328(2.083) (*r* ^2^ = 0.446, *df* = 152, *F* = 121, *P*<0.0001).

The relationship between absolute body depth (BD), mouth width (MW) estimates and total length (TL) fitted on linear regression equations (Table 1). ANCOVA analyses showed significant differences between the regression slopes of the body parts (*df* = 4, *F* = 4.988, *P*<0.0001), suggesting that absolute body depth increases faster than mouth width. The significant differences between the regression slopes of the mouth width estimates were due to the increase in the mouth width extension (MWE) as fish grew larger (Figure 2).

**Table 1:** Relationship of morphological parts of barramundi (15–140 mm TL).

Relationships	Equations	*r^2^*	*df*	*F*	*p* intercept	*p* slopes
	Absolute measures (mm)					
MWc vs TL	MWc = 0.091(0.001)^c^TL+0.850(0.034)	0.986	359	25,181	<0.0001	<0.0001
MWcmax vs TL	MWcmax = 0.093(0.001)^c^TL+1.508(0.071)	0.998	21	9,620	<0.0001	<0.0001
MWo vs TL	MWo = 0.155(0.001)^b^TL+0.462(0.117)	0.988	153	12,873	<0.0001	<0.0001
MWomax vs TL	MWomax = 0.157(0.001)^b^TL+1.067(0.134)	0.999	16	11.708	<0.0001	<0.0001
BD vs TL	BD = 0.255(0.001)^a^TL+0.483(0.063)	0.994	367	57,299	<0.0001	<0.0001
	Relative measures (% body parts)					
MWc vs MWE	MWE = −10.355(0.608)MWc+163.997(6.443)	0.657	152	284.86	<0.0001	<0.0001
MWo vs MWE	MWE = 1.259(1.196)MWo+34.324(19.439)	0.007	152	1.109	0.079	0.294
MWc vs MWo	MWc = 0.583(0.081)MWo+1.079(1.319)	0.255	152	51.711	0.415	<0.0001

**Absolute measures (mm)**

MWc: closed mouth width;

MWcmax: maximum closed mouth width;

MWo: opened mouth width;

MWomax: maximum opened mouth width;

BD: body depth;

TL: total length.

**Relative measures (% body parts)**

MWc: closed mouth width (%TL);

MWo: opened mouth width (%TL);

MWE: mouth width extension (% MWc).

A marked inter-individual variability was observed for all morphological variables. Estimates for both opened and closed mouth widths presented a consistent variability during the juvenile phase (Figure 1). Inter-individual variability was also observed for MWE, varying consistently at about 30% (±15%) for the whole range of fish size (Figure 2). The positive correlation between MWc and MWo (*r* = 0.505, *n* = 153, *P*<0.0001; Table 1) and the non-correlation between MWo and MWE (*r* = 0.085, *n* = 153, *P* = 0.294; Table 1) indicated that the MWo was more affected by the MWc than by the MWE. In contrast, the negative correlation between MWc and MWE (*r* = −0.811, *n* = 153, *P*<0.0001; Table 1) indicated that the highest MWE (Figure 2) were associated with the smallest MWc (Table 1). Therefore, the maximum values of MWc and MWo were used to develop specific models to reflect polyphenism in mouth width.

**Figure 3:**
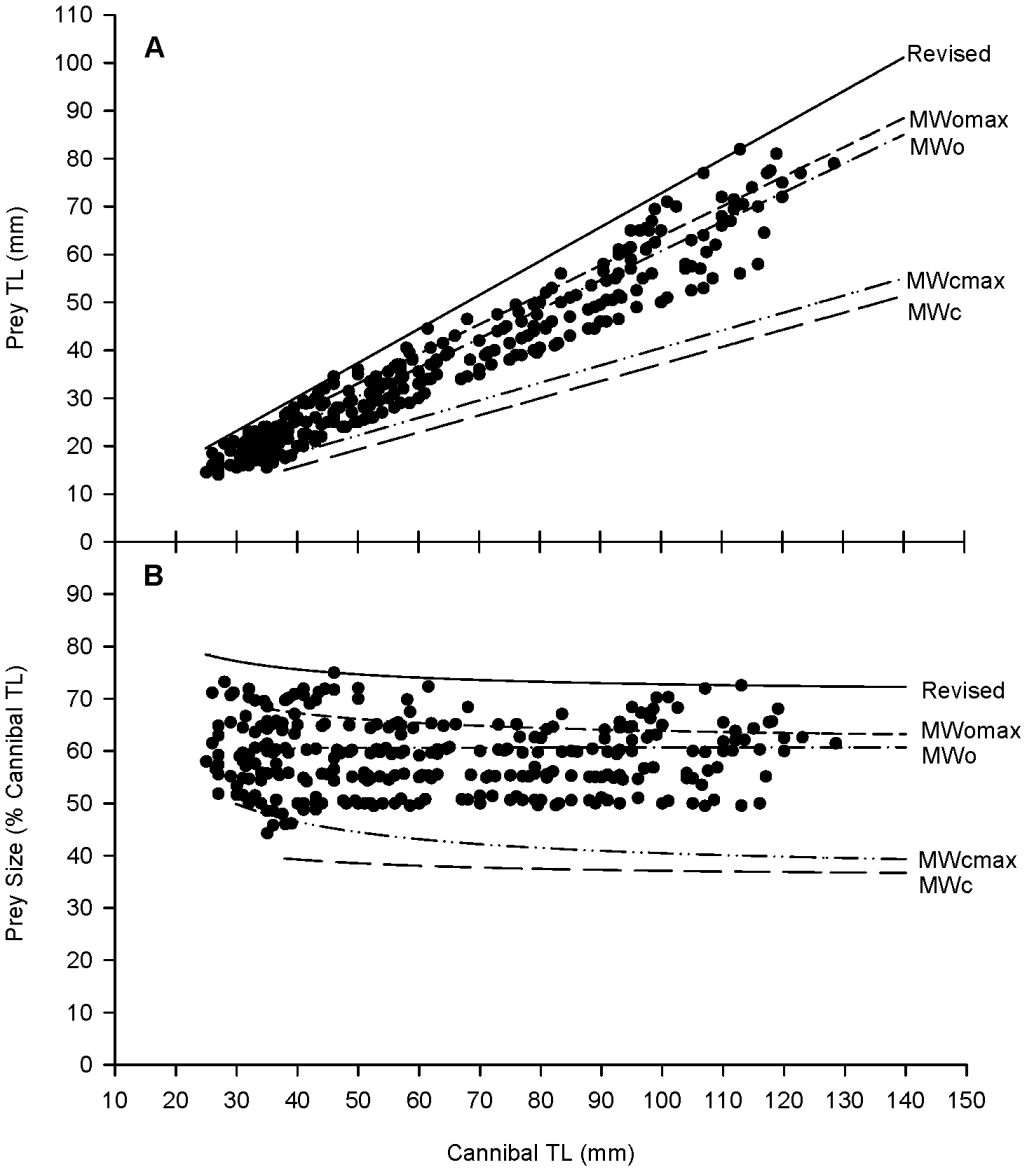
Maximum conspecific prey size for barramundi cannibals. Prey size in panel A is expressed as prey TL, mm, and in panel B expressed as % of cannibal TL. Regression lines include (1) the maximum size of prey ingested (“Revised” in filled circles on the top) and (2) model predictions on prey size based on closed mouth width (MWc), maximum closed mouth width (MWcmax), opened mouth width (MWo), and maximum opened mouth width (MWomax).

Assuming that a TL_cannibal_ could swallow a TL_prey_ if the BD_prey_ was equal to or smaller than the MW_cannibal_, the maximum conspecific prey size for cannibalistic barramundi was predicted by simple linear regression (Table 2). All models predicted that the maximum prey TL increased with increasing cannibal TL (Figure 3A). However, when expressed as a proportion of cannibal TL, the models showed a slightly declining trend in the size of maximum prey as cannibal TL increased (Figure 3B). The closed mouth width (MWc) model predicted that the maximum prey size decreased from 40 to 37% or from 50 to 39% of cannibal TL considering the maximum values (MWcmax), for cannibals of 30–140 mm TL. The maximum prey size remained constant at 61% of the cannibal TL when the model was based on the opened mouth width (MWo). However, when considering maximum opened mouth width (MWomax) the model predicted a decreasing trend from 68 to 63% of cannibal TL, for a similar size range of cannibals. Such decreasing tendencies as cannibals grew larger were related to the slightly fast increase in body depth comparing with the mouth width (Table 1).

**Table 2:** Prediction of prey size (TL_prey_, mm) from cannibal size (TL_cannibal_, mm) based on different predictive model equations for cannibalistic barramundi (30–140 mm TL).

Models	Equations
MWc	TL_prey_ = 0.3569TL_cannibal_+1.4392
MWcmax	TL_prey_ = 0.3647TL_cannibal_+4.0196
MWo	TL_prey_ = 0.6078TL_cannibal_−0.0824
MWomax	TL_prey_ = 0.6157TL_cannibal_+2.2902
Revised	TL_prey_ = 0.709TL_cannibal_+1.8881

MWc: closed mouth width;

MWcmax: maximum closed mouth width;

MWo: opened mouth width;

MWomax: maximum opened mouth width;

Revised: the model based on the empirical observations on the maximum size of ingested prey.

### Cannibal challenge

In total, 495 prey-cannibal pairs were tested using 102 potential cannibals from 25 to 131 mm TL. There was no substantial variation of the prey size during the pairing periods. In those cases when predation did not occur, the final prey size was −50–1.40% of the initial prey size as the percent of cannibal TL (*n* = 55). In all potential cannibals challenged, 75% became true cannibals ingesting at least one conspecific prey. These cannibals consumed 61.6% of the total number of prey while dead prey on the bottom accounted for 20.2%. Four cases of suffocation were observed during the trials where cannibals died with the prey stuck in mouth. In addition, three half-ingestion events were observed in this study, where the cannibals predigested half of the prey and discarded the other half. Interestingly, in all these cannibalistic events, prey sizes were 65% of cannibal TL. When the prey size was firstly offered at 58.36±5.37% cannibal TL, 25% of the large fish tested did not become cannibals, but the other 75% of the large fish became cannibals when the prey size firstly offered was 50.77±2.57% cannibal TL (T-test; df = 100; *P*<0.0001).

The results of the cannibal challenged with prey showed that cannibals were able to ingest a conspecific prey larger than the size that all models could predict (Figure 3A, B). For instance, according to the models based on MWc, MWcmax, MWo or MWomax, a cannibal of 106.50 mm TL could ingest a prey of 39, 43, 65 or 68 mm TL (37, 40, 61 or 64% of cannibal TL), respectively. Results from the cannibal challenging trial showed that identical sized cannibals could ingest a conspecific prey of 77 mm TL (72% of cannibal TL). Thus, according to empirical observations, cannibals of 25–131 mm TL could ingest the prey of 78–72% of cannibal TL, respectively. Such reduction in the maximum prey size is a result of a faster growth of the body depth in relation to the mouth size (Table 1). The increase in mouth width extension as fish grew larger (Figure 2) would compensate the part of negative allometric growth of the mouth width.

## Discussion

A model by Parazo et al. [15] predicted that cannibalistic barramundi of 10–50 mm TL (total length) can ingest a maximum conspecific prey size of 67–61% of cannibal TL, respectively. However, the empirical results in the present study showed that barramundi cannibals (25–131 mm TL) could ingest conspecific prey of 78–72% of cannibal TL, respectively. All predictive models using morphological traits considering the alternative assumption of cannibalistic polyphenism underestimate the maximum prey size that a cannibal can possibly ingest.

All successfully cannibalistic events in the present study were orientated by head being sucked in first and cannibals ingesting the whole prey. Moreover, cannibalistic barramundi ingested their prey horizontally, making the size of mouth width become the limiting factor for prey ingestion. Thus, using the closed mouth width (MWc) as an independent factor, the predictive model shows a maximum prey size of 40–37% cannibal TL, for the cannibals of 30–140 mm TL, respectively. Alternatively, when the model was developed with the opened mouth width (MWo), it predicts that a cannibal can ingest a maximum prey of 61% of the cannibal TL, which is almost double the size that the previous model predicted. Our model prediction is in accordance with that by Parazo et al. [15] who predicted a maximum prey size of 67–61% of cannibal TL, when cannibals were 10–50 mm TL, respectively, based on mouth size as the distance from the dorsal to the ventral boundary of the mouth opened. Whatever the case was, when predictive models were compared with the empirical results in this study, the models underestimate the maximum conspecific prey size that cannibals can ingest. Similar conclusions were drawn by Qin and Fast [21] on snakehead *Channa striatus* and Johnson and Post [22] on largemouth bass *Micropterus salmoides* when their predictive models were validated with empirical data.

In the present study, despite the inter-individual variability on mouth width and the capacity of mouth width extension, the models based on maximum values of the closed (MWcmax) and opened mouth widths (MWomax) underestimate the maximum ingestible capacity of cannibalistic individuals. Nevertheless, the model using MWomax predicts a slightly higher cannibalistic capacity than the model using MWo, which is closer to the empirical observation. The high inter-individual variability on MWE indicates marked polyphenism in the mouth extension capacity. However, as a negative correlation was detected between MWE and MWc, polyphenic MWE seems to be present in order to compensate the morphological disadvantages of fish with smallest MWc, but not for fish with larger MWc. As a result, the polyphenic trait of the MWo, which obviously represents the maximum predation capacity of cannibalistic barramundi, is rather a result of larger mouth width than higher mouth extension capacity. The polyphenic trait of a mouth provides not only a cannibalistic advantage, but a feeding advantage on other food. Thus, polyphenism should be considered when assessing feeding ecology of piscivorous fish species in general.

Allometric growth of the mouth is common in fish species and together with size heterogeneity it can determine the dynamic of complete cannibalism in fish [13], [24]. Previous observations on barramundi feeding showed that the onset of complete cannibalism mainly occurs after metamorphosis, when fish are being weaned to inert diets [13], [31]. In the present study, mouth width showed slower growth than the body depth. As both variables set the morphological boundary for complete cannibalism, both predictive and revised models show a decreasing trend on the maximum ingestible prey size as barramundi grow larger. As a result, cannibalism in barramundi is more likely to occur in early juvenile than during latter stages, which agrees with the findings on cannibalism by Otterå and Folkvord [24] for *G. morhua*, and Qin and Fast [21] for *C. striatus*.

Morphological constrains are not the only cause of a general reduction trend on cannibalism rate as fish grow larger. Cannibalistic fish usually prefer smaller prey as reported in *P. djambal* [23] and *Pseudoplatystoma punctifer* [32]. Thus, once smaller prey are succumbed to cannibalism, reducing the size heterogeneity of the population [13], cannibals are forced to move up to larger prey, which may not be energetically profitable as preying on smaller prey since such a size shift may represent an increase in pursuit and handling time and reduce energy gain per capture attempt [33]–[37]. In aquaculture where plenty of inert food of high energetic content is available, cannibals may choose to abandon a cannibalistic diet because such diet is not profitable anymore. In contrast, if cannibalistic individuals do enjoy growth advantages over siblings feeding on alternative diets, as observed in the Amazonian catfish *Pseudoplatystoma punctifer* [32], cannibalism will hardly become to an end as the higher growth rate of cannibals may compensate the morphological constraints as fish get larger. On the other hand, if alternative inert food is supplied accordingly, non-cannibalistic individuals may achieve more competitive growth rates [32] and growth beyond the prey spectrum of the cannibals [13]. Further studies should assess the dynamics of intracohort cannibalism in barramundi when alternative inert diet is applied at different developmental stages.

The cannibal challenge experiment was purposely designed in a small scale aiming to maximise the propensity of cannibalism. Small enclosures were used to limit escape ability of small prey and large cannibals were individually stocked, previously acclimated and deprived of alternative food, which is similar to the designs by Sogard and Olla [25] Johnson and Post [22], Hseu et al. [20] and Baras et al. [23]. In this experiment, 75% of the prey available to cannibals were ingested proving that the environment was appropriate to provoke cannibalism. Furthermore, the pairing period was defined as four days, a similar period used by Qin and Fast [21], which was assumed to be short enough to avoid significant behavioural and physiological changes in cannibals and prey, but long enough to promote maximum hunger for cannibals. In addition, cannibalistic events were orientated towards the same prey size (% cannibal TL) offered since prey and cannibals presented similar growth rates during the course of the 4-days paring period. Interestingly, barramundi could become cannibals when the first prey sizes were <50% of their predator body length, but the fish could never become a cannibal when the first prey was >58% of the cannibal size. This may indicate that, once all cannibals are removed from a barramundi population, the size difference of 50% can be a safe margin to avoid the emergence of new cannibals. Furthermore, once a fish had experienced as a cannibal, this fish would use the full morphological capacity to ingest a prey, even though the size of prey may exceed the model prediction. Challenging cannibals with an increasing prey size in the absence of alternative food may have stimulated the phenotypic plasticity in the mouth apparatus, such as hypertrophied jaw musculature [2], [8] resulting in greater predation capacity when compared with the predictive models based on fish samples taken from a fish population where food was present and cannibalism was not stimulated.

Unsuccessful cannibalistic events, such as suffocation and half-ingested prey were recorded during the cannibal challenge experiment. Previous studies have used suffocation events as a reference of the maximum prey size limit for cannibals [38], [39]. In the present study, unsuccessful cannibalistic events occurred when the prey size was 65% of cannibal TL, which is below the upper size limit determined by the revised model. However, when compared with the predictive model based on the opened mouth width (MWo), prey sizes were slightly larger than the model predicted. Therefore, it seems that those unsuccessful cannibalistic events were performed by hunger-motivated individuals to cannibalize a larger prey they could possibly handle. In addition, dead prey individuals were occasionally observed on the bottom of the tank. In most of these cases, cannibals resumed predation when a live prey of similar size was re-offered, suggesting that such event did not represent the maximum prey size they can ingest and they are probably associated with cannibal's difficulties to handle the prey or prey's abilities to escape from predation acts. Whatever the case, all these events can account for fingerling mortality leading to significant losses in fingerling production.

In summary, this study provides a new approach to predict cannibalistic events in fish under an aquaculture situation and offers recommendation on criteria for size grading practices. In order to reduce the incidence of intracohort cannibalism in a barramundi population, no conspecific prey smaller than 78–72% of cannibal TL should co-inhabit with cannibals from 30 to 140 mm TL, respectively. Furthermore, once all cannibals were removed from the population through size sorting, a size difference of 50% should be set as a threshold to avoid the emergence of new cannibals. Predictive models based on mouth width and body depth of a population average underestimate the maximum prey size for cannibalistic barramundi. However, when polyphenism was considered on measuring of the opened mouth width, the model became closer to the reality, suggesting that when predicting the upper prey size limit for complete cannibalism, the assumption of cannibalistic polyphenism must be considered to keep a safe margin and avoid significant losses due to cannibalistic mortality.

Some unsettled issues still exist. It is still unclear whether the fish with polyphenic mouth size are always consuming the largest prey and it is also uncertain whether these cannibals make the most of their prey's energy. Presumably, a large mouth facilitates handling and increases capture success, but energetic benefit of being a cannibal needs further investigation. In aquaculture, we recommend putting aside those fish that are cannibals because they could have broader mouth dimensions than others, and if this trait is heritable, it can complicate rearing in the future.
